# Prevalence of cardiovascular morbidities in Myanmar

**DOI:** 10.1186/s13104-017-2422-2

**Published:** 2017-02-15

**Authors:** Ko Ko Zaw, Nwe Nwe, Su Su Hlaing

**Affiliations:** 1 Department of Medical Research, Ministry of Health and Sports, No.5, Ziwaka Road, Dagon Township, Yangon, 11191 Myanmar; 2 Department of Medical Services, Yangon General Hospital, Ministry of Health and Sports, Yangon, Myanmar; 3 Department of Human Resources for Health, University of Public Health, Ministry of Health and Sports, Yangon, Myanmar

**Keywords:** Cardiovascular diseases, CVD, Myanmar, Prevalence, Morbidities

## Abstract

**Background:**

Cardiovascular diseases (CVDs) are now in a rising trend in South East Asia including Myanmar due to increase in major cardiovascular risk factors in both urban and rural areas, such as smoking, obesity and diabetes mellitus. It is necessary to determine CVD morbidities in Myanmar for planning of prevention and control activities for CVDs. The cross-sectional household survey was conducted in 2012 with 600 people aged 40 years and above in four townships (Kyauk-Tan, Mawlamyaing, Pathein and Pyay) and used face-to-face interview with standard questionnaire [Rose Angina Questionnaire and Questionnaire by European Cardiovascular Indicators Surveillance Set (EUROCISS) Research Group] to determine the level of reported CVD morbidities in adult population.

**Results:**

Age of the study population ranged from 40 to 99 years with the mean age of 56 years. Seventy-one percent of the study population was women. Nine percent of the study population have suffered from angina according to Rose Angina Questionnaire. Prevalence of possible heart attack, stroke and heart failure was 7.5, 1.5 and 2.8%. Prevalence of hypertension was 51%.

**Conclusion:**

The CVD morbidities are high. There is a need for strengthening prevention and control activities of CVDs.

## Background

Communicable diseases were the main causes of death around the world for centuries. Major epidemics frequently limited life expectancy. After the Second World War, with medical research achievements in terms of vaccination, antibiotics and improvement of life conditions, non communicable diseases (NCDs) started causing major problems in industrialized countries [1].

Cardiovascular diseases (CVDs) is the major category of noncommunicable diseases. CVDs include diseases of the heart, vascular diseases of the brain and diseases of blood vessels. The first half of the twentieth century saw an emerging epidemic of cardiovascular diseases as a result of industrialization, urbanization, increased prosperity in the developed countries. Therefore CVDs are often thought to be diseases of the rich nations. In fact, CVDs is globalized to all parts of the world. Over the past 20 years, deaths from CVDs have been declining in high-income countries, but have increased at a fast rate in low- and middle-income countries (LMIC). CVDs now have a major impact not only on developed nations but also on low and middle income countries, where it accounts for nearly 30% of all deaths [2].

CVDs remains the leading cause of death in the world, far outstripping deaths due to malaria, human immunodeficiency virus/acquired immunodeficiency syndrome (HIV/AIDS), and tuberculosis [3]. Nearly half of the 36 million deaths due to noncommunicable diseases (NCDs) are caused by CVDs [4]. The percentage of premature deaths from CVDs ranges from 4% in high-income countries to 42% in low-income countries, leading to growing inequalities in the occurrence and outcome of CVDs between countries and populations.

The increased prevalence of risk factors for CVDs and related chronic diseases in developing countries, including tobacco use, unhealthy dietary changes, reduced physical activity, increasing blood lipids, and hypertension, reflects significant global changes in behavior and lifestyle. These changes now threaten once-low-risk regions, a shift that is accelerated by industrialization, urbanization, and globalization. The potentially devastating effects of these trends are magnified by a deleterious economic impact on nations and households, where poverty can be both a contributing cause and a consequence of chronic diseases [2].

CVDs is now in a rising trend in South East Asia including Myanmar due to increase in major cardiovascular risk factors in both urban and rural areas, such as smoking, obesity and diabetes mellitus.

In 2003–2004, Diabetes Project has conducted a survey on prevalence of diabetes mellitus and risk factors for major NCDs in Yangon Region using the World Health Organization Stepwise Approach to Surveillance of Non-communicable Disease (WHO STEPS) methodology [5]. In 2009 a national survey on NCD risk factors was done using the WHO STEPS methodology [6]. These surveys focused only on major risk factors of NCDs and did not cover knowledge, attitudes and morbidities of NCDs.

CVD Project of Myanmar conducted the survey on CVD risk factors in 2003 in Myanmar [7]. It was dealt with risk factors and knowledge of CVDs. To update the information from previous studies with more detailed information on current status of knowledge, attitudes, risk factors and morbidities of CVDs in Myanmar, CVD Project of Myanmar conducted a survey named “Cardiovascular Survey in Myanmar (2013): knowledge, attitude, risk factors and morbidities” [8].

This paper aims to present information on levels of selected cardiovascular morbidities (angina pectoris, myocardial infarction, stroke and heart failure) in four study townships, using data from this survey.

The findings of this survey are expected to provide information for situation analysis of cardiovascular health in the community, designing community-based interventions for cardiovascular health including health education and development of information, education and communication (IEC) materials for cardiovascular health.

## Methods

The household-based cross-sectional design was conducted with a representative sample of the adult population aged 40 years and above in four townships: Kyauk-Tan township from Yangon Region, Mawlamyaing township from Mon State, Pathein township from Ayeyarwaddy Region and Pyay township from Bago Region. People living in institutions and in very poor health were excluded from the survey. Kyauk-Tan township has 32 wards with the total population of 132,765. Mawlamyaing township, has 28 wards and 13 village tracts wards with the total population of 289,388. Pathein township has 15 wards and 40 village tracts with the total population of 287,071. Pyay township has 10 wards and 55 village tracts with the total population of 251,643.

Required sample size was calculated, using the following formula [9] with prevalence of possible myocardial infarction as the main variable.

$$\begin{aligned}N &= Number \, of \, geographical \, regions \\ & \quad \times \left( {1/1 - Non \text{-}respondent \, rate} \right) \, \times (Z_{a}^{2} \times \, P \, \left( {1 - P} \right)/e^{2} ) \end{aligned}$$

Prevalence of possible myocardial infarction (P) is assumed to be 6.7% [10]. Alpha error is set at 5%; so *Z*_α_ is 1.96. Margin of error (*e*) is set at 4%. Non-response rate is estimated to be 5%. Number of geographical regions is four. The required sample size for the whole survey turned out to be approximately 632 which is rounded up to 660 respondents for convenient allocation of the sample into clusters.

The multistage cluster sampling method was used to recruit respondents. From the list of wards and villages in each township, 15 wards and villages were selected according to probability proportional to size of the population (PPS). From each ward or village selected, 11 households were selected, using systematic sampling. From each selected household, one eligible person was recruited into the study. If more than one eligible person were present in a selected household, only one eligible person was selected randomly.

Data collection was completed during January 2013 to April 2013. The trained interviewers collected data on morbidities of selected cardiovascular diseases (angina pectoris, myocardial infarction, stroke and heart failure), using the structured questionnaire. The questions on angina pectoris and myocardial infarction were based on Rose Angina Questionnaire [11]. The questions on stroke and heart failure were developed based on recommended questions by EUROCISS Research Group [12]. These questions were translated into Myanmar language and pretested and revised into the final questionnaire.

After each interview, the interviewers checked for completeness and consistency of questionnaire. The data quality and validity of measurements were overseen by the supervisors. After cleaning of data, data entry was done using Epi data software. Finally, data were analyzed using STATA to describe the main outcome variables as percent and 95% confidence interval by sexes and age groups (40–59 years and 60+ years).

From the Rose Angina Questionnaire, the respondents were classified as ever having had angina symptoms based on standard criteria. Based on the Rose Angina Questionnaire, informants were classified as having had a possible myocardial infarction (heart attack) if they reported having ever had an attack of severe pain across the front of the chest, lasting for half an hour or more. This is referred to in this report as ‘possible myocardial infarction’ (irrespective of medical diagnosis).

This study has been approved by Ethics Review Committee of Department of Medical Research. We obtained informed written consent from every participant to conduct the interview with him or her, and to publish their data.

## Results

### Background characteristics

Of 660 eligible adults aged 40 years and above invited to participate in the study, 600 adults (90.9%) actually participated in the study. Two-thirds of the respondent was aged 40–59 years age group and there was female preponderance in the study population. The majority of the respondents were married and had education of middle school or lower.

### Morbidities of CVDs

Tables 1, 2, 3, 4 display prevalence of selected cardiovascular diseases (angina pectoris, myocardial infarction, stroke and heart failure). All these tables refer to ever having the condition.

**Table 1:** Prevalence of angina pectoris (by Rose Angina Questionnaire)

Age group (years)	Men			Women			Both sexes			*P* value*
	n	Angina pectoris (%)	95% CI	n	Angina pectoris (%)	95% CI	n	Angina pectoris (%)	95% CI	
40–59	95	4.2	[0.1, 8.3]	292	10.6	[7.1, 14.2]	387	9.0	[6.2, 11.9]	0.059
60+	77	7.8	[1.7, 14.0]	136	8.8	[4.0, 13.7]	213	8.5	[4.7, 12.2]	0.79
All ages	172	5.80	[2.3, 9.3]	428	10.0	[7.2, 10.0]	600	8.8	[6.6, 11.1]	0.098

[95% confidence interval]

* P value for the comparison between men and women of the same age group

**Table 2:** Prevalence of possible myocardial infarction (Rose Angina Questionnaire)

Age group (years)	Men			Women			Both sexes			*P* value*
	n	Possible MI (%)	95% CI	n	Possible MI (%)	95% CI	n	Possible MI (%)	95% CI	
40–59	95	8.4	[2.7, 14.1]	292	10.3	[6.8, 13.8]	387	9.8	[6.8, 12.8]	0.59
60+	77	2.6	[0, 6.2]	136	8.8	[4.0, 13.7]	213	6.7	[3.2, 9.9]	0.078
All ages	172	5.8	[2.3, 9.3]	428	9.8	[7.0, 12.6]	600	8.7	[6.4, 10.9]	0.11

[95% confidence interval]

* P value for the comparison between men and women of the same age group

**Table 3:** Prevalence of stroke

Age group (years)	Men			Women			Both sexes			*P* value*
	n	Stroke (%)	95% CI	n	Stroke (%)	95% CI	n	Stroke (%)	95% CI	
40–59	95	6.3	[1.3, 11.3]	292	4.5	[6.8, 13.8]	387	4.9	[2.7, 7.1]	0.46
60+	77	10.4	[3.4, 17.4]	136	9.6	[4.6, 14.6]	213	9.9	[5.8, 13.9]	0.84
All ages	172	8.2	[4.0, 12.3]	428	6.1	[3.8, 8.4]	600	6.7	[4.7, 8.7]	0.35

[95% confidence interval]

* P value for the comparison between men and women of the same age group

**Table 4:** Prevalence of heart failure

Age group (years)	Men			Women			Both sexes			*P* value*
	n	Heart failure (%)	95% CI	n	Heart failure (%)	95% CI	n	Heart failure (%)	95% CI	
40–59	95	5.3	[0.7, 9.8]	292	7.9	[4.8, 11.0]	387	7.2	[4.6, 9.8]	0.39
60+	77	5.2	[0.1, 10.3]	136	5.1	[1.4, 8.9]	213	5.2	[2.2, 8.2]	0.98
All ages	172	5.2	[1.9, 8.6]	428	7.0	[4.6, 9.4]	600	6.5	[4.5, 8.5]	0.42

[95% confidence interval]

* P value for the comparison between men and women of the same age group

Table 1 displays prevalence of angina pectoris from Rose Angina Questionnaire. Nearly one-tenth of the study population ever had angina pectoris in their lifetime. Prevalence of angina pectoris in the women was nearly two times higher than that in the men.

Table 2 displays prevalence of possible myocardial infarction from Rose Angina Questionnaire. Nearly one-tenth of the study population ever had possible myocardial infarction in their lifetime. Prevalence of possible myocardial infarction in the women was higher than that in the men.

Table 3 displays prevalence of stroke which was self-reported physician-diagnosed. About 7% of the study population ever had stroke in their lifetime. Prevalence of stroke in the men was higher than that in the women. Prevalence of stroke in the 60+ year age group was two times higher than that in the 40–59 year age group.

Table 4 displays prevalence of heart failure which was self-reported physician-diagnosed. About 7% of the study population ever had heart failure in their lifetime. Prevalence of heart failure in the women was higher than that in the men.

**Fig. 1:**
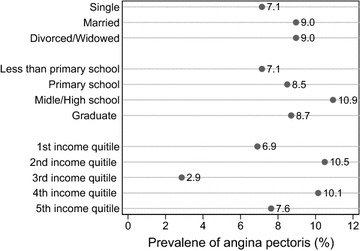
Selected socioeconomic characteristics and prevalence of angina pectoris

Figure 1 shows prevalence of angina pectoris by marital status, education level and quintiles of annual household income. The prevalence of angina pectoris was higher in married persons and divorced and widowed persons than in single persons. It increased from persons with less than primary schooling to persons with middle/high schooling and then went down to graduated persons. It has no regular pattern across annual income quintile groups. The differences in prevalence of angina pectoris were not statistically significant (at the 0.05 level) for all three socioeconomic characteristics.

**Fig. 2:**
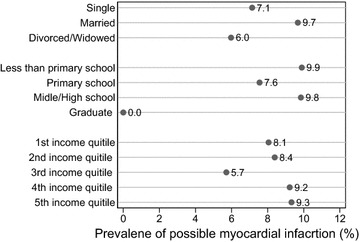
Selected socioeconomic characteristics and prevalence of possible myocardial infarction

Figure 2 shows prevalence of possible myocardial infarction by marital status, education level and quintiles of annual household income. The prevalence of possible myocardial infarction was higher in married persons than in divorced and widowed persons and single persons. It was much lower in graduate persons than in lower education levels. All income quintile groups had similar prevalence possible myocardial infarction except for 3rd income quintile group who had lower prevalence than other income quintile groups. The differences in prevalence of possible myocardial infarction were not statistically significant (at the 0.05 level) for all three socioeconomic characteristics.

**Fig. 3:**
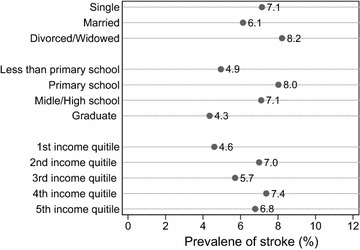
Selected socioeconomic characteristics and prevalence of stroke

Figure 3 shows prevalence of stroke by marital status, education level and quintiles of annual household income. Divorced and widowed persons had a little higher prevalence of stroke than single persons and married persons. It increased from persons with less than primary schooling to persons with primary schooling and then decreased through middle/high schooling to graduated persons. It has no regular pattern across annual income quintile groups. The differences in prevalence of stroke were not statistically significant (at the 0.05 level) for all three socioeconomic characteristics.

**Fig. 4:**
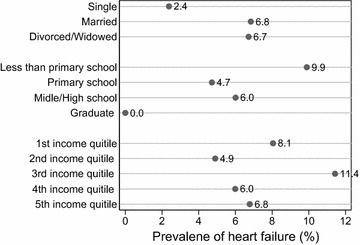
Selected socioeconomic characteristics and prevalence of heart failure

Figure 4 shows prevalence of heart failure by marital status, education level and quintiles of annual household income. The prevalence of angina pectoris was higher in married persons and divorced and widowed persons than in single persons. It was highest in persons with primary schooling and lowest in graduate persons. It has no regular pattern across annual income quintile groups. The differences in prevalence of heart failure were not statistically significant (at the 0.05 level) for all three socioeconomic characteristics.

## Discussion

The survey provided a community profile of cardiovascular morbidities among adults aged 40 years and above living in lower Myanmar. There was a female preponderance in the respondents. Most respondents were married with an education level of middle school and lower.

Nine percent of the study population had ever had angina pectoris and another 9% had ever had possible myocardial infarction. Overall prevalence of stroke and heart failure was 7%. But prevalence of above cardiovascular morbidities was higher in the women than the men except for stroke where the reverse was true.

Angina prevalence varied widely across countries, from 0.73 to 14.4% in women and from 0.76 to 15.1% in men [13]. Angina prevalence in our study population (9%) is higher than those in other Asian countries such as 2.9 in India [13], 4.95% in Sri Lanka [13], 5.7% in Nepal [14] and 3% in Bangladesh [15] but prevalence of possible myocardial infarction in our respondents (9%) is comparable to high prevalence Asian countries such as 9% in urban populations of Northern India [14].

There were some limitations in determining cardiovascular morbidities. These assessments were done, using the questionnaire, and therefore some subjectivity may influence the accuracy of morbidity assessments. The Rose Angina Questionnaire used in this survey was originally developed to identify the characteristic symptom complex known as angina in a standard way, irrespective of medical diagnosis. Its validity has been established predominantly by studies comparing the questionnaire with clinical diagnosis in men [8]. But it is a questionnaire that records symptoms in a standardized manner (chest pain relieved by rest) rather than the presence of disease. Especially in women, the questionnaire fails to distinguish coronary from noncoronary symptoms [8]. The questionnaire is also not recommended for use in older people. Anyway, presently it represents the standardized tool in field settings. The respondents only include the surviving people if they had had cardiac diseases and cannot include the people with cardiac diseases who were dead before the survey time. So these levels of cardiac morbidities are probably underestimates of these conditions.

## Conclusion

High prevalence of cardiovascular diseases in 4 study townships of Myanmar indicated the need for both facility-based care for people with cardiac illnesses and community-based prevention for cardiac health including awareness raising for the public to change behaviours to reduce cardiovascular risk factors and seek early and regular care if they are at high risk or already develop cardiovascular diseases. Both personal care and preventive initiatives should be put in place by primary health care approach, based on evidence-based interventions such as WHO’s package of essential noncommunicable disease interventions for primary health care in low-resource settings (PEN) [15].

## Abbreviations

AIDS: acquired immunodeficiency syndrome
CVDs: cardiovascular diseases CVDs
EUROCISS: European Cardiovascular Indicators Surveillance Set
HIV: human immunodeficiency virus
IEC: information, education and communication
LMIC: low- and middle-income countries
NCDs: non communicable diseases
PEN: package of essential noncommunicable disease interventions for primary health care in low-resource settings
PPS: probability proportional to size of the population
WHO STEPS: World Health Organization Stepwise Approach to Surveillance of Non-communicable Disease
